# Episode of Situated Learning to Enhance Student Engagement and Promote Deep Learning: Preliminary Results in a High School Classroom

**DOI:** 10.3389/fpsyg.2019.01415

**Published:** 2019-06-26

**Authors:** Ilaria Terrenghi, Barbara Diana, Valentino Zurloni, Pier Cesare Rivoltella, Massimiliano Elia, Marta Castañer, Oleguer Camerino, M. Teresa Anguera

**Affiliations:** ^1^“Riccardo Massa” Department of Human Sciences for Education, University of Milano-Bicocca, Milan, Italy; ^2^Catholic University of the Sacred Heart, Milan, Italy; ^3^National Institute of Physical Education of Catalonia (INEFC), University of Lleida (UdL), Lleida, Spain; ^4^Faculty of Psychology, Institute of Neurosciences, University of Barcelona, Barcelona, Spain

**Keywords:** flipped class, EAS, observation, mixed methods, qual-quan integration, engagement

## Abstract

Teaching is now experiencing a new centrality due to the fast socio-cultural transformations, the vertical growth of digital media and, therefore, the new ways children and young people learn. New paradigms and teaching methodologies are emerging to meet the new educational needs; among them, the “Episodes of Situated Learning” approach (EAS in Italian) was chosen for this study. This approach broadly refers to the “Flipped Class” model, in which the lesson structure reverses the traditional teaching/learning cycle with a positive outcome on engagement and learning. The present study aims to explore whether the EAS teaching methodology, according to literature about the Flipped Class model, has a positive outcome on student engagement, focusing on its emotional, cognitive and behavioral components. In particular, we hypothesize that the EAS teaching methodology changes teachers’ behavior in classroom, increasing their movements and body expression during the lesson. Moreover, we expect higher levels of self-efficacy and positive emotions and lower levels of perceived anxiety in teachers, thus improving students’ level of engagement. The research was conducted in a secondary school, in Milan, and includes a classroom of sixteen students and three teachers. We chose a quasi-experimental nested design, a mixed-method approach that combines the qualitative and quantitative collection and analysis of data, in order to reach, as far as possible, a holistic, effective and exhaustive representation of the studied phenomenon. Pre-post measures, including video-recording, systematic observation and questionnaires, of both students and teachers were collected during the 8 months of experimentation. This research project could foster positive outcomes for participants as well as the broader society, in which school dropout is increasing. Many authors positively associate low levels of students’ engagement to high rates of school dropout; for this reason, working on improving teaching methodologies and students’ engagement measurement, could be an effective way to enhance learning and opposing school dropout.

## Introduction

School, today, has to be restored and transformed into a “school of independence” (Mariani, 2017), where didactics have to become more experiential (Freinet, 1978), reflective, in a way that provides disciples with meaningful learning, deep and stable in time. In this perspective (Rivoltella and Rossi, 2012), schools should grant teachers the necessary conditions to implement an effective and authentic learning context, where learning individuals have the opportunity to train their skills in an active and participative environment, developing significant learning (Ausubel, 1968), with a clear transformative effect (Dewey et al., 1974).

The variables involved in developing a relevant impact on the efficacy of teaching and learning processes are many: teachers’ motivation, emotions and self-efficacy (Bandura, 1982) positively impact their perceived working satisfaction and decrease *burnout* symptoms, while also being beneficial to their disciples (Moè et al., 2010). Motivation, for example, can also be fueled by being close to motivated people. Teacher-student relationship does not only transmit certain contents, but also the desire and motivation to improve their learning experience. This consideration becomes particularly relevant when considering the fact that motivated teachers perceive themselves as capable to face specific situations, increasing, as consequence, the self-efficacy perceived by their students (Anderson et al., 1988): therefore, it is important to invest in making the “belief to be able to make it” become an ever more present belief in classrooms.

The communicative effectiveness in teaching and learning processes is not exclusively influenced by the teacher’s psychological perceptions, but it appears to be strictly and significantly correlated to the *immediacy* construct (Mehrabian, 1967; Martin and Mottet, 2011). Immediacy can be defined as the kind of communication that increases closeness between two people, and its associated behaviors express “easier approachability,” availability for communication, transmitting “warmth” and nearness (Andersen, 1985; Mazer, 2013). Several research studies have shown that, during any kind of communication, visual contact, proximity, posture facing toward the interlocutor and smiling, all constitute indicators that develop communicative intimacy, attraction and trust (Burgoon et al., 1984).

Given the above premises, the Flipped Classroom model (Baker, 2000; Lage et al., 2000), defined within *process-oriented* models (Perla, 2012), becomes significantly more relevant, especially considering the objectives of this work, since it includes all the necessary features to favor conditions useful to the development of a significant learning environment. The term “flipped” refers to the methodology, because it inverts the traditional order of the teaching/learning cycle in didactic actions. According to this method, a student approaches new information through autonomous work that usually happens at home and, once at school, the learning process sees the students involved in reworking, sharing and discussing their assignments (Bergmann and Sams, 2012).

The Flipped Classroom methodology was shown to be effective from a “student engagement” perspective (Fredricks et al., 2004), a construct that literature has shown to be significantly related to the development of learning (e.g., Connell et al., 1994; Boekarts et al., 2000). Engagement is to be intended as a multi-dimensional construct that comprehends, in its nature, diverse components that are intrinsically related. For this reason, in accordance with Fredricks et al. (2004), engagement is to be thought as a “meta-construct,” involving behavioral vectors (e.g., positive conduct, active contribution during class), emotional ones (e.g., positive emotions, sense of belonging to the institution, low anxiety levels), and cognitive ones (e.g., learning strategies and self-regulation). When considered as a synergic whole, it can describe the student experience in a comprehensive and holistic manner (Buskirk, 1961; Connell and Wellborn, 1991; Finn, 1993; Guthrie and Anderson, 1999; Wigfield and Guthrie, 2000; Stipek, 2002; Trowler, 2010).

Therefore, we can affirm that behavioral, emotional and cognitive matrixes incorporate a wide variety of constructs, highlighting the clear multi-dimensionality of engagement, as discussed by Fredricks et al. (2004), p. 65 in terms of “construct inclusiveness.”

Summing up the relationship between the quoted constructs, we can affirm that, through a Flipped Classroom planning, it is possible to foster engagement in students (e.g., Mok, 2014; Clark, 2015), making the learning process more effective and significant.

This research project takes in consideration the Episodes of Situated Learning, ESL^1^ (“Episodi di Apprendimento Situato,” EAS in italian; Rivoltella, 2013) as a relevant case study in the Italian context. The ESL model is a Teaching and Learning Activity (TLA), part of many didactic models and capable to foster meaningful learning opportunities (Rivoltella, 2017). Like the Flipped didactics, ESL can provide students the opportunity to link contents before coming into the classroom, through activities aimed at increasing interest and curiosity. Nonetheless, ESL didactics present a three-way structure, with three main work phases, each one comprising specific actions by teacher and students (Rivoltella, 2017). The first, anticipatory, phase, is focused on the student’s problem-solving ability, and gives space for discovery. In this phase, the teacher organizes the work the students will do at home, giving it an “anticipatory” function concerning the class’s contents. During the class, the teacher will provide a conceptual framework to organize the acquired contents and finally give the students a stimulus accompanying an assignment. The second phase is an operational one, where the teacher asks the group of students (or an individual) to follow an activity that will result in sharing a product made by the person/group. The third phase is dedicated to restructuring, when teachings are revised, corrected, re-formed. ESL ends each class with the teacher recalling the main concepts discussed, underlining the most important aspects to remember and correcting any misconceptions.

The objective of the ESL didactics, furthermore, is to facilitate a kind of deep learning (Ausubel et al., 1953; Gardner, 1999; Novak, 2001; Ausubel, 2004): the term denotes a learning that results by the assimilation of an experience (or new knowledge) with previous knowledge.

### The Present Study

The current study’s objective is to present preliminary results collected by observing one of the classes involved in a broader research, randomly extracted from the total sample.

The research project aims to explore and describe how ESL didactics differentiate from the traditional didactic employed by teachers. The objective is also to highlight the ESL model’s features, as well as the possible differences with traditional didactic styles, where the usual class structure involves the frontal transmission of its contents. Specifically, the study will focus on the teacher’s classroom management, on his/her didactic actions and the proxemics shown in class.

Considering what we proposed, a further objective is to create a *systematic observational grid* (Aureli and Perucchini, 2014) that would integrate dimensions and indicators corresponding to each investigated construct.

On a second level, the research aims to show how ESL didactic is an effective methodology by the perspective of student Engagement, in its behavioral, emotional and cognitive components, in line with the main theoretical evidences regarding the Flipped Classroom model. We also expect that an increase in engagement will favor a decrease in perceived anxiety, in both students and teachers.

Two hypotheses will be investigated by this study:

1. **Hp1**: The ESL model favors higher levels of scholastic Engagement in students, in all three components, and reduces their level of perceived anxiety.

1. **Hp2**: The ESL model favors higher levels of scholastic Engagement in teachers, in all three components, and reduces their level of perceived anxiety.

## Materials and Methods

The methodology implemented to reach the objectives stated and test the presented hypotheses will be mixed, combining collection and analysis of quantitative and qualitative data (Greene and Caracelli, 1997).

Specifically, we will use a nested quasi-experimental design (Creswell et al., 2003), with pre-test and post-test measurements, and the collection of different data:

- Quantitative data: observed frequencies gathered from the systematic coding of video-recordings, validated self-reports;
- Qualitative data: pen-and-paper observations, qualitative *ad hoc* questionnaires, focus groups.

The choice to adopt a mixed method is encouraged by the idea that this kind of approach can offer an effective and holistic interpretation of multi-componential constructs, such as the Engagement one, in complex environments such as the scholastic one (Anguera et al., 2012; Castañer et al., 2013; Camerino et al., 2014).

The comparison of quali-quantitative data also allows to reach better inferences, to increase data validity and the possibility to reach a superior level of comprehension of the phenomena, which could be lacking in a single-method study (Johnson and Onwuegbuzie, 2004).

### Sampling

The study was conducted at an Upper Secondary School in the province of Milan, Italy. The Institute, not far from the city center, offers two different study curricula, a “classical” one and a “linguistic” one.

Before the actual beginning of the research project, approval by the Ethical Committee of the University of Milano-Bicocca was requested and the committee expressed their consent, through sharing of *Protocol n. 324*.

The study saw the participation of 15 teachers (35.7% male and 64.3% female, average age: 41) and 5 classes (2 from the “classical” curriculum, 3 from the “linguistic” one) including a total of 101 students (26.7% male and 73.3% females; 20 students per class on average). Sample size was calculated using an expected Effect size of 0.40 (Cohen’s f: 0.40 “large”) and 0.80 Power (1–β). All teachers included in the study were observed at work with a specific class; therefore, each class was observed (both before and after teachers’ training) with three different teachers and subjects. This choice was made to guarantee that each class would be involved for an equal and balanced number of hours, according to the ESL model, and to partially control the effects deriving from teaching different subjects.

Considering inclusion and exclusion criteria, we remind that this study included role teachers, who spoke Italian as mother tongue, with a minimum of 2 h of class per group, without any experience with the ESL didactic model, nor any knowledge of its theoretical principles and modalities. Concerning the students, all recipients of the scholastic educational offer (*Piano Offerta Formativa*, POF), attending 11th and 12th grade, who expressed their consent to participating in the study and received consent by their parents, were eligible for this study.

The choice to select students from the 11th and 12th grades, excluding the 13th [last year of Secondary School (2nd grade) in Italy], was made to have a homogeneous sample and, consequentially, homogeneous data.

In accordance with the University Ethical Committee guidelines, informed consent was given via written form by all participants (students and teachers) and parents of the students.

### Instruments and Measurement

What follows is a panoramic view of the instruments and measures used in the study (for a summary see Table 1).

**TABLE 1:** Observed dependent variables and adopted instruments.

**Teachers**	**Students**
**Initial expectations**: *Ad hoc qualitative questionnaire*	**Behavioral engagement** *SEM (item 1–4) Systematic observation of video-recordings Pen-and-paper observations*
**Satisfaction and self-efficacy** *MESI (Motivations, Emotions, Strategies and Teaching*)	**Emotional engagement** *SEM (item 5–10) Pen-and-paper observations*
**State emotions** *PANAS (Positive and Negative Affect Schedule) Pen-and-paper observations*	**Cognitive engagement** *SEM (item 11–17) Pen-and-paper observations*
**State anxiety** *STAI T/S (State-Trait Anxiety Inventory) Pen-and-paper observations*	**State emotions** *PANAS (Positive and Negative Affect Schedule) Pen-and-paper observations*
**Class management, didactic actions, proxemics** *Systematic observation of video-recordings Pen-and-paper observations*	**State anxiety** *STAI T/S (State-Trait Anxiety Inventory) Pen-and-paper observations*
**Final impressions/opinions** *Ad hoc qualitative questionnaire*	**Final impressions/opinions** *Focus group*

#### Self-Report Questionnaires

##### *Ad hoc* questionnaire on initial expectations

Before the project’s beginning, teachers’ initial expectations were investigated: for this purpose, we made an *ad hoc* qualitative questionnaire, including open questions and attitude scales. This questionnaire was designed to collect qualitative information in order to better understand and interpret data from the systematic observation and the other psychometric instruments.

##### MESI

Teachers’ perceived self-efficacy was investigated through the MESI questionnaire (*Motivations*, *Emotions*, *Strategies*, and *Teaching*), built and validated in Italian by Moè et al. (2010).

##### PANAS

State emotions perceived by teachers and students during the observed class were measured by administering the PANAS (*Positive and Negative Affect* Schedule). The questionnaire, validated in Italian (Terraciano et al., 2003) asks the participant to express, on a 5-point scale, the level of intensity of each emotional state felt at the time of answering the questionnaire.

##### STAI T/S

Trait and state anxiety were measured through the STAI questionnaire (*State-Trait Anxiety Inventory*) in two versions: the first, concerning the usual levels of anxiety perceived by the participant, the second concerning anxiety perceived at the time of answering the questionnaire. This questionnaire, also validated in Italian (Pedrabissi and Santinello, 1989) as a 20-item scale, asking to indicate the degree of agreement with statements on a 4-point scale.

##### SEM

Behavioral, emotional and cognitive components of Student Engagement were investigated through a self-report questionnaire. The Student Engagement Measure (SEM) was chosen because of its three sub-scales, each one referring to one of the dimensions constituting the Engagement construct as we are considering it. The questionnaire was translated and adapted from the English version (Fredricks et al., 2011) through *back translation* and it includes 17 items (4-Behavioral engagement, 6-Emotional engagement, 7-Cognitive engagement) on a 5-point scale.

##### *Ad hoc* questionnaire on satisfaction and final impressions

As conclusion for the proposed project and once third phase measurements are finalized, we investigated opinions and final impressions by all participants. The teachers received an online questionnaire created for the purpose, comprising open and multiple-answer questions.

As for the questionnaire about initial expectations, this questionnaire was also designed to collect qualitative information for a better understanding and interpretation of data.

#### Focus Group

Opinions and impressions perceived by the students were also investigated through a final focus group, proposed to all classes involved once measurements were finalized.

Proposed activities included moments of discussion and workshop where, through active confrontation with other members of their class, participants could express their opinions based on experiential reflections.

Results from the thematic analysis of the focus group are still ongoing and will not be presented in this article.

#### Pen-and-Paper Observations

During the observation period, the researcher was present in the classroom and took note of personal impressions and events that would otherwise be excluded by the systematic observation conducted subsequently. In this case, the focus was on teachers and students alike, with no pre-established target variables.

#### Systematic Observation Grid and Coding Software

Systematic coding of target behaviors was possible thanks to the creation of a systematic observation grid (see Supplementary Material File) based on literature review during the first pilot study (cfr. par. Pilot study) through a series of context-specific trials that allowed to adapt the instrument in a way that would be effective before its actual implementation. The grid was subsequently implemented in a coding software, which made the procedure quicker and drastically reduced the margin for errors, resulting in clean datasets, immediately available for further elaboration and analysis. The details for both instruments follow:

The grid was planned so that all dimensions would be distinct, homogeneous, exhaustive and mutually exclusive: a first dimension requires for the researcher to define if the activity is “on,” or if the lesson has been interrupted; in the latter case, the researcher will select the “off” category and move to the next one. All the temporal segments judged valid, were then coded by 7 main dimensions. The first refers to lesson management and has 4 distinct categories referring to the individual or the group that are mainly managing that didactic segment and include: *teacher*, *student*, *independent group*, and *independent individual*. The second dimension refers to the main didactic actions the teacher uses in the classroom; there are 10 categories (some examples: *introduction*, *presentation*, *clarification*, *knowledge teaching*).

The coding grid also includes some dimensions referring to the teacher’s proxemics in the classroom. Three different criteria define this dimension: the first is *proxemic orientation* and requires the researcher to identify who the teacher is speaking to, for example the whole class, a group of students, a single one; the second criteria concerns the position the teacher is occupying in the room (*proxemic position*), while the third refers to transitions (*proxemic transition*).

The grid, as mentioned above, also includes two dimensions investigating the behavioral component of engagement, specifically *attention* and *active work* by students, as well as the students’ *level of participation* during the lesson. In this instance, agreement between observers is crucial, since these categories require the researcher to judge the level of attention and participation in the classroom on a low, medium and high scale.

The choice to exclude the systematic observation of behaviors belonging to the emotional and cognitive components of Engagement is mainly due to the difficulty, also found in literature review, to isolate clear and objective evidences concerning behaviors that refer to partly subjective and hardly observable dimensions.

##### Coding software

The grid was used with the support of LINCE software (Gabin et al., 2012; Hernández-Mendo et al., 2014), which made the adopted procedure much easier, i.e., systematic observation on a temporal basis (Aureli, 1997), where time divided in 30-s segments was considered as a useful sampling unit.

**FIGURE 1:**
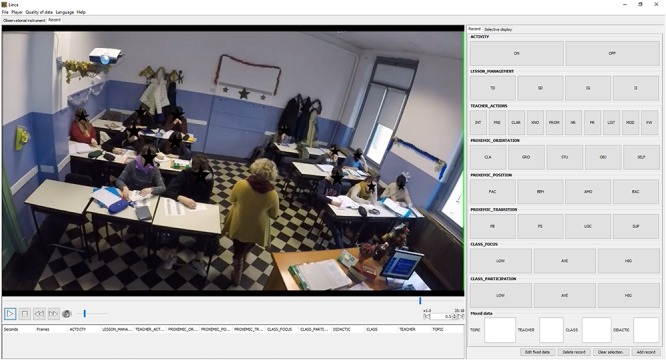
The LINCE software interface. Informed consent and authorization for the publication of this image was given via written form by all participants (students and teachers) and the students’ parents.

The software, first developed for the observation of sport performance (e.g., Cavalera et al., 2015; Diana et al., 2017), has been proven particularly suitable for the observation and coding of behaviors in general (e.g., Casarrubea et al., 2018; Diana et al., 2018), due to its clear and easy interface (see Figure 1^2^).

### Procedure and Data Collection

Measurement within subjects (before and after formation) of the dependent variables discussed above. Time was considered the independent variable in two points (T0 pre-training measurement; T1 post-training measurement).

The study was organized in four distinct and inter-dependent stages (see Table 2), from September 2017 to February 2018, for a total of 6 months. The instruments presented were used in different modalities and times, related to the study’s objectives and investigated construct. What follows is a sum up of each stage of the study, defining administration methods for each instrument.

**TABLE 2:** The research scheduling.

**September**	**October**	**November**	**December**	**January**	**February**
**2017**	**2017**	**2017**	**2017**	**2018**	**2018**
1. Pilot Study	2. Pre-training		3. EAS training	4. Post-training	
	measurements			measurements	

#### Pilot Study

A pilot study was conducted in the participating classes. Main objective of this stage was to allow participants to have a period of familiarization, in order to decrease the observer’s influence on behaviors during class. To maintain a proper ethical profile on this study, in fact, it was not possible to implement a disguised observation; during video-recordings with the researcher, while trying to render it as less intrusive as possible, the camera was not completely hidden. The familiarization period was organized in two distinct phases: the first one saw the researcher go to class with no recording devices, 1 h per class. Subsequently, the recording instruments were introduced in the classroom and the researcher explained they were turned off. Another hour per class was spent with the devices off.

The second objective for the pilot study was to prepare the experimental setting, identifying the position for the cameras and the researcher so that they would not interfere with the process. Two GoPro Hero5 cameras were used to video-record the lesson periods with external microphones for the elimination of background noise, allowing for a clear audio recording. To allow for a good visibility of the environment and classroom dynamics, cameras were placed on opposite ends of the room: the first was behind the teacher, while the second was in the back of the classroom, to have a shot of the teacher from the front. The wide-range shooting angle offered by the cameras allowed for a good total vision, without having to exclude anything from the shot. Having fixed cameras allowed to decrease the interference usually associated with a researcher moving around the room, capturing each behavior or participant involved. The experimenter, depending on the width of the room and disposition of school desks, mainly stationed in the back of the room, so as not to enter the students’ field of vision.

The third objective functional to the first stage was to define measurement instruments, specifically the coding grid used to conduct the systematic observation of target variables. The grid was built basing on related literature and was subsequently tested and revised before the actual implementation: it includes exhaustive and mutually exclusive categories for each dimension, thus allowing for a systematic coding on a temporal basis (Harrigan et al., 2008; Castañer et al., 2010, 2016).

#### Pre-post Training Measurements

Generally, measurements collected before the training class aimed to define a pre-training baseline, through the repeated measure of different dependent variables; these included, for the teachers, *initial expectations*, *teaching strategies*, *proxemics*, *classroom management, perceived self-efficacy* and, for students, *behavioral*, *emotional*, and *cognitive engagement*.

The fourth stage of the project, implemented at the end of the training course, included post-measurements, which investigated, other than the same dependent variables measured in the first stage, the final impressions of teachers and students concerning the proposed research project. In this case, objectives were to investigate the effectiveness of the training course in terms of skills learned, as well as the effectiveness of the ESL didactic model in terms of perceived school Engagement.

#### Training Course on ESL Didactic Model

The second stage of this research involved a training course, free and certified, offered to all teachers involved in the project and conducted by a CREMIT (Center for Research on Education for Media, Information and Technology) expert. The objectives of this stage were mainly to introduce the ESL didactics, explaining its theoretical bases and methodological structure and provide teachers with useful skills to plan and implement ESL didactics in class. The training course was provided on a weekly basis in a Blended mode and saw the teachers involved in four training meetings (3 h each), mainly featuring workshop activities.

During the training course, each teacher was able to build a realizable ESL program, in line with their own subjects and didactics planning, to implement later (during the post-training observations) with the assigned class. Teachers were asked to conduct their lessons according to their usual didactics style before the training course (during the pre-training observations), while they were asked to propose the ESL planned during the training for the post-training observations.

The presence of a CREMIT expert was crucial in allowing and guaranteeing every teacher to plan an ESL lesson following the correct theoretical model and its related methodological structure.

A final meeting with the teachers and students involved will be held at the end of the project. The objective will be to share the main evidences emerged and discuss impressions and feelings about the experience. This stage is currently under scheduling.

## Data Analysis and Results

All teachers who took part in the experiment were observed with their assigned class (before and after the training). Each teacher-class combination remained the same for the duration of the study, in order to allow for a stable observation and a within subjects design. Following, the preliminary results from one of the five classes involved (3 teachers and 24 students, before and after the teachers’ training), randomly extracted from the sample will be discussed.

### Systematic Observation

Encodings were carried out by two trained observers, who viewed and coded all the recorded material using a 30 s temporal unit. Inter-rater agreement was calculated through Cohen’s k (Cohen, 1960): categories included in the coding grid showed a high level of agreement, never below 0.824 (see Table 3).

**TABLE 3:** Inter-rater agreement values.

**Category**	**Agreement rate (Kappa)**
Class management	0.978
Teacher actions	0.953
Proxemic: orientation	0.974
Proxemic: position	0.968
Proxemic: transition	0.968
Class focus	0.824
Class participation	0.900

In order to maximize comparability between the two didactics modalities observed (usual teacher’s didactics vs. ESL didactics), the length of the observation period (in terms of coded time units) has to be as equivalent as possible. Therefore, we decided to randomly delete excess encodings (ESL classes, on average, lasted less than the teacher’s usual ones), obtaining 172 temporal units for each observed didactic modality (344 temporal units in total).

Kurtosis and asymmetry for all variables observed ranged between −2 and +3.56, describing a “normal” data distribution (Barbaranelli, 2007).

#### Teacher in Classroom

Class management appears to be drastically different depending on the didactic model used. As Table 4 shows, time spent handling the class by the teacher’s self significantly changes between conditions: while during a traditional lesson the teacher manages the class for almost 90% of the time (89.5%), ESL lessons see the value decrease to 8.8%, consequentially increasing space for autonomous work and moments where the students are the ones managing class.

**TABLE 4:** Differences in class management between usual lessons and EAS lessons.

**Usual lesson**		**Frequency**	**Percentage**	**% valid**
Valid	Teacher	154	89.5	***89.5***
	Student/s	6	3.5	3.5
	Group Work	11	6.4	***6.4***
	Individual Work	1	0.6	0.6
	Total	172	100.0	100.0
Missing	System	/	/	
Total		172	100.0	

**EAS lesson**		**Frequency**	**Percentage**	**% valid**

Valid	Teacher	15	8.7	***8.8***
	Student/s	59	34.3	34.5
	Group Work	97	56.4	***56.7***
	Individual Work	/	/	/
	Total	171	99.4	100.0
Missing	System	1	0.6	
Total		172	100.0	

*Bold values represent the most relevant results.*

Considering the didactic actions, data shows how, while usual didactics see the teacher presenting new content to the class for more than half the time (58.7%), ESL lessons show no sign of this kind of intervention, with a related increase in the number of times the teacher is listening to the students (their requests, reflections, sharing of their work with the class group, etc.) and clarifying their doubts. These two didactic actions were coded, respectively in 34.9 and 19.2% of cases (see Table 5).

**TABLE 5:** Difference in teacher actions between usual lessons and EAS lessons.

**Usual lesson**		**Frequency**	**Percentage**	**% valid**
Valid	Introduction	7	4.1	4.1
	Presentation	**101**	**58.7**	**58.7**
	*Clarification*	*5*	*2.9*	*2.9*
	Knowledge testing	41	23.8	23.8
	Providing materials	/	/	/
	Negative reinforcement	6	2.3	2.3
	Positive reinforcement	/	/	/
	*Listening*	*5*	*2.9*	*2.9*
	Moderating debate	/	/	/
	Personal work	9	5.2	5.2
	Total	172	100.0	100.0
Missing	System	/	/	/
Total		172	100.0	100.0

**EAS lesson**		**Frequency**	**Percentage**	**% valid**

Valid	Introduction	12	7.0	7.0
	Presentation	/	/	/
	*Clarification*	33	19.2	***19.3***
	Knowledge testing	/	/	/
	Providing materials	4	2.3	2.3
	Negative reinforcement	/	/	/
	Positive reinforcement	/	/	/
	*Listening*	60	34.9	***35.1***
	Moderating debate	/	/	/
	Personal work	62	36.0	36.3
	Total	171	99.4	100.0
Missing	System	1	0.6	
Total		172	100.0	
				

*Bold values represent the most relevant results.*

Taking the teacher’s proxemics in consideration, in its three variants, it is interesting to note how the teacher, during a usual lesson, mostly addresses the class (79.1%), occupying frontal space from the students’ perspective, between the teacher’s desk and the blackboard.

During ESL lessons, the teacher addresses more frequently specific groups of students and increases didactic moments of nearness to the students, in the middle of the room where school desks are usually placed (see Tables 6, 7).

**TABLE 6:** Proxemics differences between usual lessons and EAS lessons _orientation.

**Usual lesson**		**Frequency**	**Percentage**	**% valid**
Valid	Class	154	79.1	***79.8***
	Group	/	/	/
	Student	10	5.8	5.8
	Object	1	0.6	0.6
	Self	7	4.1	4.1
	Total	172	100.0	100.0
Missing	System	/	/	
Total		172	100.0	

**EAS lesson**		**Frequency**	**Percentage**	**% valid**

Valid	Class	22	12.8	12.9
	Group	86	50.0	50.3
	Student	1	0.6	0.6
	Object	/	/	/
	Self	62	36.0	36.3
	Total	171	99.4	100.0
Missing	System	1	0.6	
Total		172	172	

*Bold values represent the most relevant results.*

**TABLE 7:** Proxemics differences between usual lessons and EAS lessons_position.

**Usual lesson**		**Frequency**	**Percentage**	**% valid**
Valid	Facing	159	92.4	92.4
	Behind	1	0.6	0.6
	In the middle	9	5.2	5.2
	Giving the back	3	1.7	1.7
	Total	172	100.0	100.0
Missing	System	/	/	
Total		172	100.0	

**EAS lesson**		**Frequency**	**Percentage**	**% valid**

Valid	Frontal	135	78.5	***78.90***
	Behind	/	/	/
	In the middle	31	18.0	***18.0***
	Giving the back	5	2.9	2.9
	Total	171	99.4	100.0
Missing	System	1	0.6	
Total		172	100.0	

*Bold values represent the most relevant results.*

Table 8 shows proxemic transitions by the teacher, which do not suggest a significant change between the two styles.

**TABLE 8:** Proxemic differences between usual lessons and EAS lessons_transition.

**Usual lesson**		**Frequency**	**Percentage**	**% valid**
Valid	Standing	43	25.0	25.0
	Sitting	123	71.5	71.5
	Walking	5	2.9	2.9
	Support	1	0.6	0.6
	Total	172	100.0	100.0
Missing	System	/	/	
Total		172	100.0	

**EAS lesson**		**Frequency**	**Percentage**	**% valid**

Valid	Standing	37	21.5	***21.6***
	Sitting	126	73.3	73.7
	Walking	1	0.6	***0.6***
	Support	7	4.1	4.1
	Total	171	99.4	100.0
Missing	System	1	0.6	
Total		172	100.0	

*Bold values represent the most relevant results.*

#### Students in Classroom

Students’ attention during ESL lessons improves significantly: moments when most of the class is actively focused on the lesson’s contents increase by 54.7%, while moments when attention is judged as low decrease by almost 40% (see Table 9).

**TABLE 9:** Differences in attention and active work between usual lessons and EAS lessons.

**Usual lesson**		**Frequency**	**Percentage**	**% valid**
Valid	Low focus	67	39.0	39.0
	Average focus	96	55.8	55.8
	High focus	9	5.2	5.2
	Total	172	100.0	100.0
Missing	System	/	/	
Total		172	100.0	

**EAS lesson**		**Frequency**	**Percentage**	**% valid**

Valid	Low focus	/	/	/
	Average focus	68	39.5	21.7
	High focus	103	59.9	***76.7***
	Total	171	99.4	100.0
Missing	System	1	0.6	
Total		172	100.0	

*Bold values represent the most relevant results.*

Active participation by the students also seems to be increased during ESL lessons, judging by the indicators values: during usual lessons, the class is completely involved in 4.7% of cases, while ESL lessons see that percentage increase by over 50%, and moments when the students’ participation was judged as low decreased by 55.3% (see Table 10).

**TABLE 10:** Differences in active participation between usual lessons and EAS lessons.

**Usual lesson**		**Frequency**	**Percentage**	**% valid**
Valid	Low partic.	163	94.8	94.8
	Average partic.	1	0.6	0.6
	High partic.	8	4.7	4.7
	Total	172	100.0	100.0
Missing	System	/	/	
Total		172	100.0	

**EAS lesson**		**Frequency**	**Percentage**	**% valid**

Valid	Low partic.	68	39.5	***39.8***
	Average partic.	/	/	/
	High partic.	103	59.5	***60.2***
	Total	171	99.4	100.0
Missing	System	1	0.6	
Total		172	100.0	

*Bold values represent the most relevant results.*

### Psychometric Questionnaires: Teachers

To accurately describe and evaluate each considered variable, we conducted paired samples *t*-tests. Perceived self-efficacy by the teachers (MESI) did not show any significant change (*p* = 0.427); no significant change for perceived emotions (PANAS) during teaching activities was found as well (*p* = 0.303).

Perceived anxiety (STAI S) during usual lessons and ESL lessons appears to be slightly higher during ESL (+1.6 points). These changes were not found to be significant (*p* = 0.701).

### Psychometric Questionnaires: Students

All questionnaires were analyzed through paired samples *t*-tests. The SEM (Student Engagement Measure) highlights positive results for each of the three components constituting the Engagement construct: scores on each sub-scale significantly increase for both Behavioral, Emotional, and Cognitive Engagement.

Table 11 summarizes scores obtained and significance for each result. The level of total perceived Engagement increases to 11 points.

**TABLE 11:** Student Engagement (SEM), pre- and post.

**CLASS 3**		***Mean***	***Means difference***	***p***
Pair 1	**Eng. Tot. Pre**	50.7917	–11.75	0.000
	**Eng Tot. Post**	62.5417		
Pair 2	Behavioral Pre	15.3333	–2.54	0.000
	Behavioral Post	17.8750		
Pair 3	Emotional Pre	18.1667	–4.54	0.000
	Emotional Post	22.7083		
Pair 4	Cognitive Pre	17.2917	–6.67	0.000
	Cognitive Post	21.9583		
	Cognitive Post	21.3125		

Finally, considering emotions felt by the students (PANAS) during the lesson, it is possible to notice a significant change regarding positive emotions in the classroom. Considering all questionnaires administered, we see how students perceive higher levels of positive emotions and lower levels of negative ones during ESL lessons; the former see an increase of about 11 points, while the latter decrease by 4 points. *T*-tests show a significant change only on the first (positive emotions, see Table 12).

**TABLE 12:** Emotions (PANAS) perceived by students, pre- and post.

		**Standard**	**Std. Error**	**Means**			
**Perceived Emotions**	**Mean**	**deviation**	**difference**	**difference**	**t**	**gl**	***p***
Emozioni Positive Pre	76.300	14.46874	4.57542	–11.18496	–3.477	9	0.007
Emozioni Positive Post	87.500	15.36410	4.85855				
Emozioni Negative Pre	41.400	13.99365	4.42518	4.10000	–1.204	9	0.259
Emozioni Negative Post	37.300	9.32202	2.94788				

Through paired samples *t*-tests analysis, it was possible to notice how perceived anxiety in students (STAI S) during usual lessons was higher than the one perceived during ESL lessons. Results are not statistically significant (see Table 13).

**TABLE 13:** Perceived anxiety (STAI S) by students, pre- and post.

		**Standard**	**Std. Error**	**Means**			
**ANXIETY**	**Mean**	**deviation**	**difference**	**difference**	**t**	**gl**	***p***
Anxiety Pre	39.2667	9.72689	3.07591	3.40000	1.402	9	0.194
Anxiety Post	35.8667	5.23332	1.65492				

### Expectations and Final Reflections by Teachers: *Ad hoc* Questionnaires

#### Expectations

The initial expectations questionnaire administered to the teachers highlighted their curiosity and interest toward the project addressing them; adjectives used by the three teachers were “curiosity” and “interest.” The reflection concerning strengths and weaknesses in the proposed approach highlighted, on one side, a desire for the training to be interesting and stimulating, and the preoccupation it would be too tiresome or even useless on the other. In this case, teachers used adjectives such as “stimulating” and “interesting,” as well as “tiresome,” “time-consuming,” and “useless.”

Examining the scores given by teachers to the sentences offered by the questionnaire, wide expectations are detected regarding the acquisition of new teaching strategies and new digital instruments in support of teaching. Low expectations were expressed concerning acquisition of new manuals for professional education.

#### Final Reflections

As stated above, after the video-recording stage, all teachers were administered with another questionnaire, mainly aiming at collecting opinions and subjective impressions that could contribute to the interpretation of quantitative data. What follows is a quick summary of each of the seven questions comprised in the questionnaire, highlighting thematic cores extracted by reading the analyzed answers.

The first two questions asked, in general, about the main strengths and weaknesses in ESL didactics found by teachers.

On a fully shared perception emerging, is that of a stimulating teaching way, both for teachers and students (“*it forced me to think of a relationship between a subject and contemporary suggestion*”), as well as an engaging experience (“*students are free to participate in a more direct manner*,” “*involving them in first person*”).

Among the critical elements that were found, the most important seems to be its time-consuming nature (“*time-consuming, because it requires more hour than a usual lesson on the subject to prepare*”).

The third question asked teachers if, according to their own personal experience, the ESL approach facilitated participation and involvement in students. The teachers responded positively in all cases.

The fourth question asked teachers to reflect on the class atmosphere perceived during the ESL lesson, detailing a change when compared to the usual climate. In this case, teachers reported an improvement of their relationship with and cooperation between students (“*the climate in the classroom is different: in the beginning, everyone is fairly skeptical, because of the “new approach” they have to confront with, but during the practical stage a good work and cooperative climate establishes between students*”).

The two final questions addressed emotions and feelings by the teachers when they were managing their teaching actions. The first question asked to state the main emotional states perceived (“*calm*” and “*comfortable*” were the main answers), while the second question addressed personal satisfaction with the work done (“*yes, I am very satisfied*”).

In conclusion, a multiple-choice question addressing future intentions on the use of ESL didactics asked if the teachers would use the ESL method again. The three teachers included in this article have expressed their intention to use ESLS didactics again in 100% of cases, choosing the answer “*yes, surely*.”

## Discussion

The observational grid used to encode video-recordings represented the main result for this work. The objective was to build the observation instrument and, secondarily, to verify whether its implementation would produce coherent results. From this perspective, the instrument presents a good internal reliability, allowing replication and decreasing the number of errors due to misinterpretation or subjective beliefs in observers (Hintze et al., 2002). The choice of a closed set of categories (exhaustive and mutually exclusive) and behaviors that occur within them, forces the researcher to a previous and clear conceptual analysis, other than a better pre-definition of categories, therefore producing a better result in terms of validity and an advantage in terms of reliability (Aureli, 1997; Castañer et al., 2010, 2016). The implementation of the coding grid in LINCE software has also allowed to collect wide-ranging datasets with a variety of immediately available data for different kinds of analysis, optimizing research times.

The more explorative objective of this work was to describe how ESL didactics differentiate from the traditional didactic acted by teachers and highlight the peculiarities of the ESL model; preliminary evidence shows how the ESL approach involves the teacher and the student in an active construction of didactic action, in a different way from the habitual practice (Rivoltella, 2014). Going further than the mere exposition of didactic contents, the teacher becomes more involved in the relationship with the students: not only explaining things but also listening to them, their questions, clarifying doubts, expressing appreciation toward the students, asking questions and encouraging active participation, getting closer by decreasing the usual proxemic distance separating the teacher from the class. The evidence underline how an ESL teacher can transmit availability for communication and psychological immediacy, indicators of communicative effectiveness and positive linked with students’ interest and involvement on the learning process (Andersen, 1985).

Furthermore, class management is defined as a shared dynamic, where the students themselves co-construct the teaching action: students are less likely to perceive themselves as passive recipients of an educational offer and more likely to actively live the learning process (Dewey, 2004). In this perspective, the student is the first actor of the teaching play and the teacher does not limit to apply pre-ordered didactic models; educational practice become a continuous search for the most effective ways to shape teaching and learning processes through the construction of a shared meaning. Results shows how ESL didactics seems to encourage teachers to occupy the central space of the classroom, and move between desks, more than what usually happens with a positive impact on students’ attention.

The first hypothesis stated that the ESL model favors higher levels of scholastic Engagement in students, in all three components, and reduces their level of perceived anxiety). Preliminary results from this study show a clear and significant positive correlation between ESL modality and Student Engagement in all its components; the decrease in anxiety was not found to be significant.

Active and participative involvement of the students emerges univocally from different kinds of data and analyses. Students have expressed a positive judgment of the experience, describing ESL as a stimulating didactic modality, requiring them to work in first person and, for this, allowing to foster engagement on a behavioral perspective.

From the emotional point of view, students experienced more, and more intense, positive emotions, and a higher level of satisfaction perceived during the ESL; this evidence is confirmed focusing on the STAI item “I feel satisfied,” which responses were widely more positive after the ESL lessons. In fact, a satisfied student is the one who perceives to have a personal role in what he/she does, having to choose the best strategy to reach established objectives (Moè et al., 2010) as it happens in every-day life, where the individual is called to be a protagonist and has to take adaptive decisions (Goldberg, 2005).

The ESL didactics seems effective on a Cognitive Engagement perspective as well; results from the SEM questionnaire show that students employ a higher level of effort, and adopt cognitive strategies, to foster effective learning (for example by trying to watch TV shows dealing with the topics brought in class, or by reading “extra” books to deepen what has been learned). These dynamics create the necessary conditions for the student to be strategically involved, fostering significant learning (Ausubel, 1968) and the establishment of relationships between the old and the new meanings. In fact, students report themselves trying to regulate their attention, creating connections between different information, actively monitoring their comprehension level through self-evaluation and all of these are effective strategies.

The first hypothesis is therefore partially accepted, since the decrease in anxiety was not found to be significant.

The second hypothesis stated that the ESL model favors higher levels of scholastic Engagement in teachers, in all three components, and reduces their level of perceived anxiety. Preliminary results from this study show that Engagement levels seem to not change significantly; analyses highlighted a slight increase in perceived anxiety and a decrease in the self-efficacy judgment.

The teachers probably perceived the project proposal as new and challenging, provoking a higher psychological activation. As underlined by in-depth qualitative information, teachers define the planning of an ESL lesson as highly time-consuming and hardly repeatable during the course of the school year (mostly because of curricula structure). Perceived self-efficacy, referred to the belief a person has in him/herself and their capabilities, represents a relatively stable component and it is possible that a better assessment could be obtained through a longitudinal study (e.g., Caprara et al., 2011) and/or a different measurement tool.

The second hypothesis is therefore rejected.

## Conclusion, Limits, Future Perspectives

Strength points of this work are mainly related to its multi-disciplinary nature, emerged from the heterogeneity of the investigated constructs and the mixed research design, which called for the collection of different kinds of data and the application of different instruments and methods of analysis. This project has included participants from a Secondary School (II grade), increasing the relatively scarce evidence to be found in literature regarding this specific school period. A positive fallout interested the teachers and students involved: the formers were active recipients of a free and certified training, the latter experienced a diverse educational offer, more stimulating and meaningful.

This research could have a positive impact on a broad level: school Engagement is focal for students but also something for a teacher to be considered when planning or evaluating their actions. The correct measurement of this variable could bring an improvement in awareness about teaching practices and could foster deep learning in students. Moreover, ESL didactics seem to represent an effective methodology in terms of school Engagement; defining engagement as an impactful co-variable on dropout levels (Fall and Roberts, 2012), the systematic introduction in the school *curricula* of Teaching and Learning Actions like the ESL model could help in preventing school dispersion.

The main limits of this work are to be found in the sample size and extraction. Furthermore, repeated measurements and follow-ups would have been useful to evaluate the results stability on the long term, considering the continuously changing nature of the environment, where students and teachers represent interested participants of a cycle of growth and evolution. This research concerned state variables as well as stable constructs (such as perceived self-efficacy in teachers), thus it could be useful to replicate measurements over time and to test other instruments for a better measurement. New instruments could be proposed, for example, to monitor change in teachers’ emotions and perceived anxiety, important to be considered and measured for future studies.

Academic research could help in improving and suggesting new transformative pathways. On this note, it would be interesting to further improve and test the multi-method measurement protocol used in this work, with the objective of a multi-language validation (both in Italian and English first); this would improve the validity of the results and could allow international collaborations and studies.

Future actions will include further analyses on the entire corpus of data that will provide more evidences; furthermore, the feedback meetings (currently ongoing) will be crucial for the design and development of new research lines and studies on this matter and context.

## Data Availability

The datasets generated for this study are available on request to the corresponding author.

## Ethics Statement

This study was carried out in accordance with the recommendations and guidelines of the “University of Milano-Bicocca Ethical Committee,” with written informed consent from all subjects. All subjects gave written informed consent in accordance with the Declaration of Helsinki. The protocol was approved by the “University of Milano-Bicocca Ethical Committee.” with the Protocol Number 324.

## Author Contributions

IT, BD, and VZ contributed to the method development, study designing, data analysis, and manuscript writing. PR contributed to study designing and data acquisition and coding. ME contributed to data analysis. MC and OC contributed to method development and data analysis. MA contributed to method development and manuscript writing. All authors made suggestions and critical reviews to the initial draft and contributed to its improvement until reaching the final manuscript, which was read and approved by all authors.

## Conflict of Interest Statement

The authors declare that the research was conducted in the absence of any commercial or financial relationships that could be construed as a potential conflict of interest.

## Footnotes

1 The ESL acronym has not to be confused with the same mentioned in Packler’s work (1959): in this case ”ELS” indicates a didactic model theorized by Rivoltella (2013) in the Italian context.

2 Informed consent and authorization for the publication of this image was given via written form by all participants (students and teachers) and the students’ parents.
