# Supplementary material for: Episode of Situated Learning to Enhance Student Engagement and Promote Deep Learning: Preliminary Results in a High School Classroom
Source: Front Psychol. 2019 Jun 26;10:1415. doi: 10.3389/fpsyg.2019.01415 (PMC6607896; doi:10.3389/fpsyg.2019.01415)
Supplement: Supplementary file 1 [file Data_Sheet_1.pdf]

Attachment 1

Systematic coding grid

**DIMENSIONI** (DIMENSION)	**CATEGORIE (**ANALITYCAL CATEGORIZATION)	**COD.**	**INDICATORI** (DESCRIPTION)	**REF.**
**ATTIVITA'** (ACTIVITY)	**ON** (ONGOING)	ON	The selected temporal segment can be coded	Aureli, 1997; Hintze, Volpe & Shapiro, 2002
	**OFF** (OFFGOING)	OFF	The selected temporal segment cannot be coded (the lesson has been interrupted by an external interference: school announcements, presence of other teachers or school operators, etc.)	
**GESTIONE D'AULA** (CLASS MANAGEMENT)	**INSEGNANTE** (TEACHER_DRIVEN)	TD	The teacher is managing the classroom (presenting didactic content, introducing an activity, moderating a debate...), while the class is listening	Rivoltella, 2013; Mariani, 2017
	**STUDENTE/I** (STUDENT_DRIVEN)	SD	One or more students are managing the classroom (presenting a report, group work...), the teacher is listening	
	**LAVORO INDIPENDENTE DI GRUPPO** (INDIPENDENT_GROUP)	IG	Students are busy with group activities (research, exercise, creating artifacts...)	
	**LAVORO INDIPENDENTE INDIVIDUALE** (INDIPENDENT_INDIVIDUAL)	II	Students are busy with individual activities (exercise, research...)	
**AZIONI DIDATTICHE** (TEACHER ACTIONS)	**INTRODUZIONE** INTRODUCTION	INT	The teacher introduces the lesson ("today we will discuss/plan...") or the activity to be conducted (by explaining rules, modalities and objectives)<br>The teacher indicates a new exercise to the students "on page 20, the exercise we will carry out..."<br>The teacher divides the class in groups to prepare for the following work phase<br>The teacher prepares work spaces<br>The teacher calls and/or introduces a group that will present their work	Tacconi, 2015; Carenzio, Triacca & Rivoltella, 2014
	**SPIEGAZIONE** PRESENTATION	PRE	The teacher introduces a didactic content	

	**CHIARIMENTO**<br>CLARIFICATION	CLAR	The teacher answers questions or doubts concerning the lesson or the activity's contents<br>The teacher makes sure the class/group/student has clearly understood the concept, demands or times for the activity<br>The teacher makes sure the student or group is working correctly<br>The teacher clarifies the functions or structure of didactic material (website, photocopies, books)	
	**VERIFICA APPRENDIMENTI**<br>KNOWLEDGE_TEXTING	KNO	The teacher verifies the class or student's knowledge through questions, homework correction, practice...	
	**DISTRIBUZIONE MATERIALE**<br>(PROVIDE_MATERIAL)	PROM	The teacher provides the class with work/study materials, proposes or distributes didactic cards<br>The teacher prepares or turns on the PC for group work and/or indicates websites to be explored<br>The teacher withdraws the distributed materials	
	**RINFORZO NEGATIVO**<br>(NEGATIVE_REINFORCE)	NR	The teacher requests order, calls out disturbing students, remarks or calls for attention	
	**RINFORZO POSITIVO**<br>(POSITIVE_REINFORCE)	PR	The teacher enhances and reinforces positive behaviors, expresses appreciation, calls for attention by using positive ironic communication	
	**ASCOLTO**<br>(LISTENING_TO)	LIST	The teacher listens to and/or observes the work by single students or groups.<br>The teacher listens to the exposition of a work or content by the students<br>The teacher listens to a comment or intervention by a student	
	**CONDUZIONE DIBATTITO**<br>(MODERATING_DEBATE)	MOD	The teacher involves the class in a discussion where students have to express their personal opinion on a subject, content, or activity	
	**LAVORO PERSONALE**<br>(PERSONAL_WORK)	PW	The teacher fills in the class registry, or carries out activities not related to the lesson contents	
**PROSSEMICA: ORIENTAMENTO**<br>(PROXEMIC: ORIENTATION)	**CLASSE**<br>(CLASS)	CLA	The teacher addresses the whole class	Castañer, Camerino, Anguera & Jonsson, 2016
	**GRUPPO**<br>(GROUP)	GRO	The teacher addresses a group of students	
	**STUDENTE**<br>(STUDENT)	STU	The teacher addresses a single student	
	**OGGETTO**<br>(OBJECT)	OBJ	The teacher attends to the didactic content object of discussion (reads a passage, watches a video)	
	**Sé STESSO**<br>(SELF)	SELF	The teacher is engaged in personal activities	

**PROSSEMICA: POSIZIONE** (PROXEMIC: POSITION)	**DI FRONTE** (FACING)	FAC	The teacher is facing the students, in the frontal portion of the classroom (near desk/blackboard)	
	**DIETRO** (BEHIND)	BEH	The teacher is behind the students, in the back of the room	
	**IN MEZZO** (AMONG)	AMO	The teacher is in the center of the room, among desks	
	**DI SPALLE** (BACK_TURNED)	BAC	The teacher is giving his back to the students (e.g., writing on blackboard)	
**PROSSEMICA: TRANSIZIONE** (PROXEMIC: TRANSITION)	**IN PIEDI FERMO** (FIXED_BIPEDAL_POSTURE)	FB	The teacher is standing and not moving (or just a few steps, no more than 3)	
	**SEDUTO** (FIXED_SEATED_POSTURE)	FS	The teacher is sitting	
	**IN MOVIMENTO** (LOCOMOTION)	LOC	The teacher is walking, moving through space	
	**SUPPORTO** (SUPPORT)	SUP	The teacher is still, leaning on a support (desk, wall, etc.)	
**ATTENZIONE/LAVORO ATTIVO** (CLASS FOCUS)	**BASSO** (LOW)	LOW	Students are mostly distracted, few actively work and show attention. Even fewer are focused and are working on the lesson's contents	Blatchford, 2003; Blatchford, Bassett, Brown, 2011; Skinner, Kindermann, & Furrer, 2009; Sink & Spencer, 2005; Anderson, Hamilton & Hattie, 2004
	**MEDIO** (AVERAGE)	AVE	Some students are attentive, actively working, and are carrying out activities related to the lesson's contents. Others are not working and are distracted (about 50/50).	
	**ALTO** (HIGH)	HIG	Most students are attentive, focused on the didactic contents offered. Few are distracted and are not actively working (or carrying our unrelated activities)	
**PARTECIPAZIONE** (CLASS PARTICIPATION)	**BASSO** (LOW)	LOW	Students are mostly passive. None or few students participate (or try to, by raising their hand or trying to speak) in the activity, asking or answering questions, proposing coherent reflections or discussing with their classmates	
	**MEDIO** (AVERAGE)	AVE	Some students participate and interact (or try to) in a coherent manner, asking or answering questions, proposing reflections (50/50)	
	**ALTO** (HIGH)	HIG	Most students are actively participating (or trying to) in the activity, answering or asking questions, offering personal inherent reflections	
